# Sunscreens Cause Coral Bleaching by Promoting Viral Infections

**DOI:** 10.1289/ehp.10966

**Published:** 2008-01-03

**Authors:** Roberto Danovaro, Lucia Bongiorni, Cinzia Corinaldesi, Donato Giovannelli, Elisabetta Damiani, Paola Astolfi, Lucedio Greci, Antonio Pusceddu

**Affiliations:** 1 Department of Marine Sciences; 2 Institute of Biochemistry and; 3 Department of Chemical Sciences and Technologies, Faculty of Science, Polytechnic University of the Marche, Ancona, Italy

**Keywords:** bleaching, corals, sunscreens, UV filters, viruses

## Abstract

**Background:**

Coral bleaching (i.e., the release of coral symbiotic zooxanthellae) has negative impacts on biodiversity and functioning of reef ecosystems and their production of goods and services. This increasing world-wide phenomenon is associated with temperature anomalies, high irradiance, pollution, and bacterial diseases. Recently, it has been demonstrated that personal care products, including sunscreens, have an impact on aquatic organisms similar to that of other contaminants.

**Objectives:**

Our goal was to evaluate the potential impact of sunscreen ingredients on hard corals and their symbiotic algae.

**Methods:**

*In situ* and laboratory experiments were conducted in several tropical regions (the Atlantic, Indian, and Pacific Oceans, and the Red Sea) by supplementing coral branches with aliquots of sunscreens and common ultraviolet filters contained in sunscreen formula. Zooxanthellae were checked for viral infection by epifluorescence and transmission electron microscopy analyses.

**Results:**

Sunscreens cause the rapid and complete bleaching of hard corals, even at extremely low concentrations. The effect of sunscreens is due to organic ultraviolet filters, which are able to induce the lytic viral cycle in symbiotic zooxanthellae with latent infections.

**Conclusions:**

We conclude that sunscreens, by promoting viral infection, potentially play an important role in coral bleaching in areas prone to high levels of recreational use by humans.

Coral reefs are among the most biologically productive and diverse ecosystems in the world, representing hot spots of marine biodiversity, and directly sustaining half a billion people (Moberg and Folke 1999; Wilkinson 2004). Approximately 60% of coral reefs are currently threatened by several natural and anthropogenic impacts (Hughes et al. 2003; Pandolfi et al. 2003). Over the last 20 years, massive coral bleaching (i.e., loss of symbiotic zooxanthellae hosted within scleractinian corals) has increased dramatically, both in frequency and spatial extent (Hoegh-Guldberg 1999; Hughes et al. 2003; Knowlton 2001). This phenomenon has been associated with positive temperature anomalies, excess ultraviolet (UV) radiation or altered available photo-synthetic radiation, and presence of bacterial pathogens and pollutants (Brown et al. 2000; Bruno et al. 2007; Douglas 2003; Glynn 1996; Jones 2004).

Production and consumption of personal care and cosmetic sun products are increasing worldwide, reaching unexpected levels, with potentially important consequences on environmental contamination. The release of these products is also linked with the rapid expansion of tourism in marine coastal areas (Wilkinson 2004). Chemical compounds contained in sunscreens and other personal care products have been demonstrated to reach detectable levels in both fresh and sea-water systems (Daughton and Ternes 1999; Giokas et al. 2007). These compounds are expected to be potentially harmful for the environment; hence, the use of sunscreen products is now banned in a few popular tourist destinations, for example, in marine ecoparks in Mexico, and in some semi-enclosed transitional systems (Xcaret 2007; Xel-ha 2007). Because sunscreens are lipophilic, their UV filters can bioaccumulate in aquatic animals (Giokas et al. 2007) and cause effects similar to those reported for other xenobiotic compounds (Balmer et al. 2005; Daughton and Ternes 1999). Paraben preservatives and some UV absorbers contained in sunscreens have estrogenic activity (Daughton and Ternes 1999; Schlumpf et al. 2004). In addition it has been demonstrated that several sunscreen agents may undergo photodegradation, resulting in the transformation of these agents into toxic by-products (Giokas et al. 2007, and literature therein).

Recently, it has also been demonstrated that sunscreens have an impact on marine bacterioplankton (Danovaro and Corinaldesi 2003), but there is no scientific evidence for their impact on coral reefs.

To evaluate the potential impact of sun-screen ingredients on hard corals and their symbiotic algae, we conducted several independent *in situ* studies with the addition of different concentrations of sunscreens to different species of *Acropora* (one of the most common hard-coral genus), *Stylophora pistillata,* and *Millepora complanata.* These studies were performed from 2003 to 2007 in different areas of the world, including the Celebes Sea (Pacific Ocean), the Caribbean Sea (Atlantic Ocean), and the Andaman Sea and the Red Sea (Indian Ocean).

## Materials and Methods

### Study areas and experimental design

*In situ* experiments were conducted in four coral reef areas: Siladen, Celebes Sea (Indonesia, Pacific Ocean); Akumal, Caribbean Sea (Mexico, Atlantic Ocean); Phuket, Andaman Sea (Thailand, Indian Ocean), and Ras Mohammed, Red Sea (Egypt, Indian Ocean). Nubbins of *Acropora* spp. (~ 3–6 cm) were collected, washed with virus-free seawater filtered onto 0.02-μm membranes (Anotop syringe filters; Whatman, Springfield Mill, UK), immersed in polyethylene Whirl-pack bags (Nasco, Fort Atkinson, WI, USA) filled with 2 L virus-free seawater, and incubated *in situ*. Additional experiments were also performed with other hard coral genera: *S. pistillata* and *M. complanata*. Replicate sets containing nubbins from different colonies *(n* = 3, including more than 300 polyps each) were supplemented with aliquots of sunscreens (at final quantities of 10, 33, 50, and 100 μL/L seawater) and compared with untreated systems (used as controls). Corals were incubated at the same depth of donor colonies at *in situ* temperature (Table 1). During two experiments conducted in the Red Sea and in the Andaman Sea, we tested the effects on coral bleaching of the same chemical filters and preservatives contained in the sunscreen formula of different brands (Tables 1 and 2). Subsamples (50 mL) of seawater surrounding coral nubbins were collected at 12-hr intervals and fixed in 3% glutaraldehyde for subsequent analyses (i.e., zooxanthellae counts and transmission electron microscopy, TEM). Additional sea-water samples were immediately processed without any preservation for viruslike particles counts. At the end of the experiments, samples of coral tissue were fixed in 3% glutaraldehyde and stored at 4°C for zooxanthellae count and TEM.

### Quantification of bleaching

To quantify the levels of coral bleaching (Siebeck et al. 2006), we performed a colorimetric analysis on digital photographs of corals taken at the beginning of the experiments and after various times of treatment with sunscreen and organic UV filters. Photographs were taken under identical illumination with a Canon PowerShot A620 digital camera (Canon Inc., Tokyo, Japan) with a scale meter on the background. The photographs were successively analyzed with a photo-editing software for color composition [cyan, magenta, yellow, black (CMYK)]. Levels of bleaching were measured as the difference between the coral’s color at the beginning of the experiments and after treatments. Variations in the percentage of the different color components (CMYK) were analyzed with one-way analysis of variance (ANOVA; Table 3). To rank the bleaching effect due to the different ingredients tested, we obtained Bray–Curtis similarity matrix and multidimensional scaling analysis of the shifts in CMYK color composition of treated corals using Primer 5.0 software (Primer-E Ltd., Plymouth, UK). Bleaching rates were measured as the dissimilarity percentage in CMYK color composition between treated and control corals using the SIMPER tool of Primer 5.0 software (Primer-E Ltd).

### Analysis of zooxanthellae

Zooxanthellae were extracted from coral nubbins using a jet of artificial seawater with a WaterPick (Braun, Germany) and centrifuged (4,000 × *g*, for 10 min) to separate the algae from the host tissue. Replicate suspensions (200–500 μL) of zooxanthellae extracted from coral tissue and those released during the experiment were filtered through 2.0-μm polycarbonate filters and mounted on glass slides. Zooxanthellae were counted under a Zeiss Axioplan epifluorescence microscope (Carl Zeiss Inc., Jena, Germany; ×400 and ×1,000), and the number of cells was normalized to nubbins’ area. Based on the autofluorescence and gross cell structure, zooxanthellae released or extracted from nubbins were classified as *a*) healthy (H, brown/bright yellow color, intact zooxanthellae); *b*) pale (P, pale yellow color, vacuolated, partially degraded zooxanthellae); transparent (T, lacking pigmentations, mostly empty zooxanthellae; Mise and Hidaka 2003). Cell integrity was also examined by TEM (see below).

### Standard sunscreen UV filters for the experiments

The UV filters ethylhexyl-methoxycinnamate (OMC), octocrylene (OCT), benzophenone-3 (BZ), ethylhexylsalicylate (EHS), and the solvent propylene glycol (PG) (Table 2) were purchased from Sigma-Aldrich Co. (Milan, Italy); 4-*tert*-butyl-4-methoxydibenzoylmethane was obtained in the form of Eusolex 9020 from Merck (Darmstadt, Germany). 4-Methylbenzylidene camphor was synthesized according to Saito et al. (2004). Specifically, a mixture of *d*-camphor (10 mmol), *p-*tolualdehyde (12 mmol), and potassium *t*-butoxide (15 mmol) was refluxed in *t*-butyl alcohol (12 mL) for 5 hr. The reaction course was monitored by thin-layer chromatography using cyclohexane–ethyl acetate 8:2 as the eluant. The reaction mixture was neutralized with 5% HCl and extracted with ethyl acetate (10 mL × 3); the combined organic extracts were washed with saturated NaCl solution and dried over Na_2_SO_4_. Evaporation of the solvent and column chromatography of the crude residue on silica gel eluting with cyclohexane–ethyl acetate 8:2 gave 4-methylbenzylidene camphor as a white solid which was crystallized from hexane (70% yield). ^1^H NMR (200 MHz, CDCl_3_): δ = 0.8 (s, 3H), 0.99 (s, 3H), 1.03 (s, 3H), 1.48–1.60 (m, 2H), 1.70–1.85 (m, 1H), 2.12–2.20 (m, 1H), 2.37 (s, 3H), 3.10 (d, 1H, *J* = 4.1 Hz), 7.19 (d, 2H, *J =* 8.0 Hz). 7.21 (s, 1H), 7.38 (d, 2H, *J* = 8.0 Hz) ppm. The preservative BP (butyl paraben) was obtained through esterification of 4-hydroxybenzoic acid with butyl alcohol: 20 mmol 4-hydroxybenzoic acid was dissolved in 25 mL butyl alcohol in the presence of a catalytic amount of *p-*toluensulfonic acid (~ 2 mmol) and refluxed for 7 hr. The reaction mixture was washed with NaHCO_3_ 0.5 M and extracted with diethyl ether (25 mL × 3). The organic layer was dried over Na_2_SO_4_ and the solvent evaporated under reduced pressure. Butyl paraben was obtained with a 75% yield. ^1^H NMR (200 MHz, CDCl_3_): δ = 0.97 (t, 3H, *J* = 7.1 Hz), 1.38–1.65 (m, 2H), 1.70–1.76 (m, sH), 4.30 (t, 2H, *J* = 6.5 Hz), 6.89 (d, 2H, *J* = 8.88 Hz), 7.95 (d, 2H, *J* = 8.8 Hz) ppm. The amounts of UV filters and preservatives used in the sun-screen addition experiments were calculated on the basis of the percentage concentrations of the respective filters allowed in sunscreen formulations in both American and European markets. Hence, concentrations below the more restricted limits imposed by American regulations were used: BMDBM (2%), BZ (6%), OMC (6%), OCT (6%), EHS (5%), MBC (3%), BP (0.5%).

### Quantification of sunscreen release in seawater

To estimate the amount of UV filters and preservatives released from sunscreen formulae, 2 mg sunscreen/cm^2^ [dose recommended by the U.S. Food and Drug Administration (FDA); Poiger et al. 2004] was applied to the hands of two volunteers. The hands were then immersed in 2 L of 0.45-μm filtered seawater at 24°C for 20 min. Hands without sunscreen applications were used as controls. All experiments were repeated 3 times. The percentage of sunscreen released into the seawater was estimated by high performance liquid chromatography (HPLC) analyses on the sunscreen and seawater samples.

Some investigators suggest that the sun-screen dose recommended by the U.S. FDA is much lower than the amount actually used by tourists (Giokas et al. 2007, and literature therein); thus, the quantity of sunscreen released during a usual bath could be far higher than that estimated in this study.

### HPLC analysis of sunscreens

UV filters were extracted from 1 L seawater obtained from the sunscreen release experiment by solid-phase extraction (SPE) (C_18_ Bakerbound SPE column, 500 mg/6 mL; J.T. Baker, Phillipsburg, NJ, USA). Before extraction an internal standard, butylcinnamate (BC, Sigma-Aldrich Co.) was added to the seawater sample. The SPE column was conditioned with 10% methanol, and the sample was passed through the column at approximately 20 mL/min. The ingredients were recovered from the column using 1 mL acetonitrile. Analyses were performed on an HPLC apparatus consisting of a Varian RP-C18 column (5 μm, 250 × 4.60 mm), a 20-μL injection loop, a Varian Pro Star solvent delivery module, a Varian Star 5.0 Workstation and Varian 9050 variable wavelength UV-VIS detector (Varian Inc., Palo Alto, CA, USA). The analytes injected into the chromatograph eluted in 18 min (1 mL/min) using a linear gradient starting from solution A (methanol:acetonitrile:water:acetic acid, 55:20:24:1, vol/vol) and ending with solution B (methanol:acetonitrile:water:acetic acid, 55:40:4:1, vol/vol). UV detection was carried out at λ = 255 nm for BP and λ = 300 nm for MBC, OMC and BC. Chromatograms were analyzed with the Varian Interactive Graphics Program.

### Viral counts and infection of zooxanthellae and TEM analysis

Water samples for viral counts were processed immediately without any fixative with SYBR green and SYBR Gold staining (Shibata et al. 2006). Immediately after collection, subsamples (200 μL) of seawater surrounding coral nubbins were diluted 1:10 in prefiltered MilliQ, filtered through a 0.02-μm pore-size Anodisc filter (25-mm diameter, Al_2_O_3_; Whatman) and immediately stained with 20 μL SYBR Green I and SYBR Gold (stock solution diluted 1:20 and 1:5,000 respectively; Invitrogen, Carlsbad, CA, USA). Filters were incubated in the dark for 15 min and mounted on glass slides with a drop of 50% phosphate buffer (6.7 mM, pH 7.8) and 50% glycerol containing 0.25% ascorbic acid (Shibata et al. 2006; Helton et al. 2006; Wen et al. 2004). Slides were stored at 20°C until analysis. Counts were obtained by epifluorescence microscopy (magnification, ×1,000; Zeiss Axioplan) by examining at least 10 fields, that is, at least 200 cells or particles per replicate.

TEM analyses were conducted on decalcified corals (2% vol/vol formic acid, 4°C, 8 days. *Acropora* tissue and pellets of zooxanthellae released during the experiment were post-fixed in 1% osmium tetroxide (Sigma-Aldrich Co.), dehydrated through an increasing acetone series (25%, 50%, 75%, 100%) and embedded in an Epon–Araldite mixture (Multilab Supplies, Fetcham, UK). Ultrathin resin sections (50–70 nm) were cut with a Reichert Ultracut E microtome (Reichert, Wien, Austria). Before analysis, sections were stained with saturated uranyl acetate and 1% lead citrate and collected on 200-mesh copper/rhodium grids (Multilab Supplies).

### Estimates of release of sunscreen in reef areas

The global release of sunscreens in areas harboring coral reefs can be roughly estimated from their average daily use and the number of tourists. An average dose application of 2 mg/cm^2^ of sunscreen (dose suggested by the U.S. FDA) for a full body surface of 1.0 m^2^ results in an average usage of 20 g per application (Poiger et al. 2004). We consider a conservative measure of two daily applications per tourist traveling on a 5-day average tourist package, and a rough estimate of 78 million of tourists per year in areas hosting reefs [10% of world tourists registered in 2004; United Nations World Trade Organization (UNWTO) 2007]. Based on this calculation and on annual production of UV filters, between 16,000 and 25,000 tons of sun-screens are expected to be used in tropical countries. According to our experiment, it is estimated that at least 25% of the amount applied is washed off during swimming and bathing, accounting for a potential release of 4,000–6,000 tons/year in reef areas. Because 90% of tourists are expected to be concentrated in approximately 10% of the total reef areas, we estimated that up to 10% of the world reefs is potentially threatened by sunscreen-induced coral bleaching.

## Results and Discussion

### Coral bleaching caused by sunscreens and UV filters

**Figure 1:**
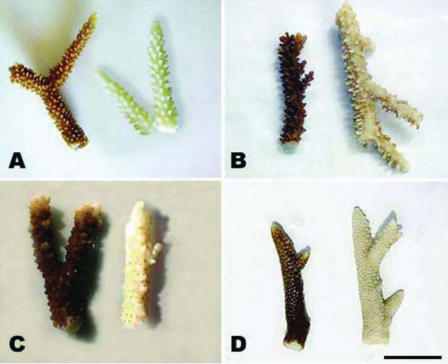
Impact of sunscreen addition on nubbins of *Acropora*. Untreated (brown) and treated (bleached) nubbins of (*A*) *Acropora cervicornis* (Caribbean Sea, Mexico); (*B*) *Acropora divaricata* (Celebes Sea, Indonesia); (*C*) *Acropora* sp. (Red Sea, Egypt); and (*D*) *Acropora intermedia* (Andaman Sea, Thailand). Images were taken within 62 hr of the start of sunscreen incubations. Scale bar = 2 cm.

**Figure 2:**
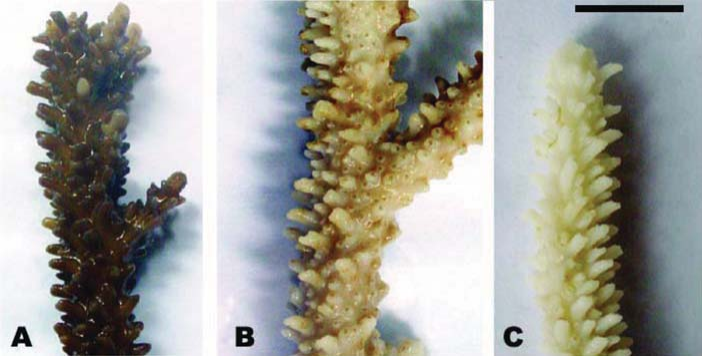
Effect of 100-μL sunscreens on *Acropora divaricata* nubbins after 24-hr incubation at various temperatures. (*A*) control; (*B*) nubbins incubated at 28°C; and (*C*) nubbins incubated at 30°C. Scale bar = 1 cm.

**Figure 3:**
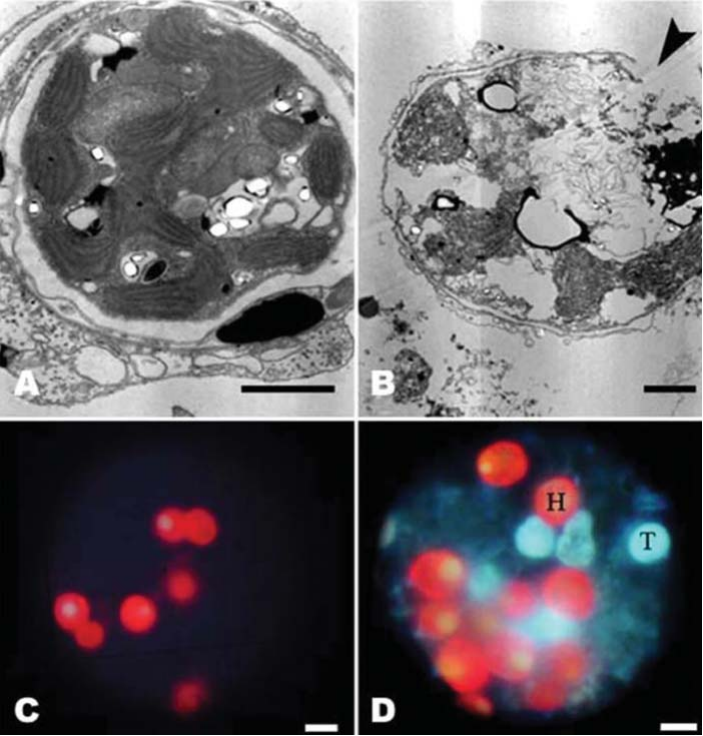
Zooxanthellae release from hard corals in control and sunscreen addition samples. (*A*) TEM images of healthy zooxanthellae (intact cell structure and membrane) in control untreated *Acropora* nubbin, and (*B*) zooxanthellae damaged by sunscreen treatment: cells appear swollen and vacuolated, without chloroplasts and double the size of the controls; the thylakoids are unpacked and dispersed inside the cells, and cell-membrane integrity is lost (arrowhead). (*C*) Autofluorescence images showing healthy (red) zooxanthellae in control sample and (*B*) some healthy (H) and damaged and partially damaged (T, transparent and pale) zooxanthellae released after sunscreen treatment. Scale bars = 2 μm (*A*, *B*) and 5 μm (*C*, *D*).

**Figure 4:**
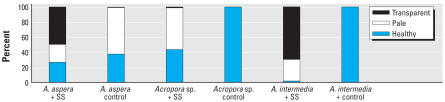
Epifluorescence microscopy analysis of the level of damage in zooxanthellae released after sun-screen (SS) addition.

In all replicates and at all sampling sites, sunscreen addition even in very low quantities (i.e., 10 μL/L) resulted in the release of large amounts of coral mucous (composed of zooxanthellae and coral tissue) within 18–48 hr, and complete bleaching of hard corals within 96 hr (Figure 1; Table 1). Different sunscreen brands, protective factors, and concentrations were compared, and all treatments caused bleaching of hard corals, although the rates of bleaching were faster when larger quantities were used (Table 1). Untreated nubbins (coral branches of 3–6 cm) used as controls did not show any change during the entire duration of the experiments (Table 1). Bleaching was faster in systems subjected to higher temperature, suggesting synergistic effects with this variable (Table 1; Figure 2). TEM and epifluorescence microscopy analyses revealed a loss of photo-synthetic pigments and membrane integrity in the zooxanthellae released from treated corals (30–98% of zooxanthellae released from *Acropora* nubbins were partially or totally damaged, appearing pale and transparent), whereas zooxanthellae membranes from untreated corals were intact (37–100% of the zooxanthellae released showed a defined shape and red fluorescing color; Figures 3 and 4). All these results indicate that sunscreens have a rapid effect on hard corals and cause bleaching by damaging the symbiotic zooxanthellae.

We tested sunscreen (10 μL/L) containing concentrations of UV filters higher than those reported in most natural environments. At the same time, the coral response to sunscreen exposure was not dose dependent, as the same effects were observed at low and high sun-screen concentrations. Therefore, we hypothesize that UV filters can have potentially negative impacts even at concentrations lower than those used in the present study.

Sunscreens typically comprise up to 20 or more chemical compounds. To identify the organic UV filters or preservatives possibly responsible for coral bleaching, seven compounds typically present in sunscreens were selected (Table 2), and additional experiments were carried out in which each single ingredient was tested on *Acropora* spp. Among the ingredients tested, butylparaben, ethylhexylmethoxycinnamate, benzophenone-3 and 4-methylbenzylidene camphor caused complete bleaching even at very low concentrations (parabens account for 0.5% of sunscreen ingredients). Conversely, all other compounds tested (i.e., octocrylene, ethylhexylsalicylate, and 4-*tert-*butyl-4-methoxydibenzoylmethane) and the solvent propylene glycol, which is also present in sunscreen formulations, had a minor effect or no effects when compared with controls (Table 1). These results suggest that sunscreens containing parabens, cinnamates, benzophenones, and camphor derivatives can contribute to hard-coral bleaching if released into natural systems.

### Amounts of sunscreen released into tropical environments and their impacts

Sunscreen product sales exceed half a billion dollars (Shaath and Shaath 2005), and it is estimated that 10,000 tons of UV filters are produced annually for the global market. According to official data of the UNWTO, it can be estimated that 10% of sunscreens produced are used in tropical areas with coral reefs (Wilkinson 2004). We estimated that, on average, about 25% of the sunscreen ingredients applied to skin are released in the water over the course of a 20-min submersion. According to these estimates, we believe that up to 10% of the world’s coral reefs would be threatened by sunscreen-induced coral bleaching.

The impact of sunscreens would be expected to be crucial in atolls and coastal coral reefs with low water renewal and strong tourist vocation. Our results provide strong scientific evidence of the potential impact of these products in tropical habitats and represent a pointer for outlining specific regulations for protecting coral reefs.

### Effect of sunscreen ingredients on viral infections

**Figure 5:**
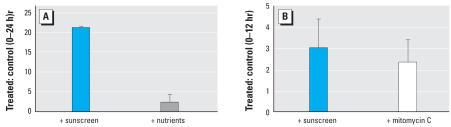
Viral enrichment factors of ambient seawater (as the ratios of viral density in treated and control samples) after the addition of sunscreen, nutrient, and mitomycin C. (*A*) Viral enrichment factor of ambient seawater within 24 hr after sunscreen and organic nutrients addition. (*B*) Viral enrichment factor of ambient seawater within 12 hr after sunscreen and mitomycin C addition. Organic nutrients (lipids, proteins, and carbohydrates) were added at concentrations equivalent to those contained in sunscreens according to Danovaro and Corinaldesi (2003). Values are ± SE.

**Figure 6:**
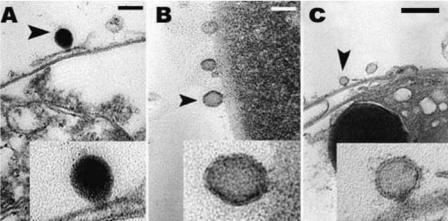
TEM images of viruslike particles (VLPs) associated with zooxanthellae released from nubbins after sunscreen treatment. (*A*, *B*) VLPs attached to zooxanthellae membranes. (*C*) Viruses attached to outer part of zooxanthellae with visible tail penetrating cell membrane. Scale bars = 100 nm (*A*, *B*); 200 nm (*C*). Arrowheads indicate sections magnified in insets.

Previous studies have demonstrated that sunscreens can significantly enhance viral production in seawater by inducing the lytic cycle in prokaryotes with lysogenic infection (equivalent to the latent infection of eukaryotes; Danovaro and Corinaldesi 2003). Here, we demonstrate that a similar phenomenon occurs also in hard corals. After the addition of sunscreens, viral abundance in seawater surrounding coral branches increased significantly, reaching values greater by a factor of 15 than in controls (Figure 5A). Because, prior to any treatment, the hard corals were carefully washed with and incubated in virus-free seawater, we conclude that the viruses encountered were released from the corals or their symbionts. Moreover, addition of organic nutrients without UV filters or preservatives did not result in coral bleaching or in a significant increase in the number of viruses in the ambient seawater (Figure 5A). Hard-coral bleaching and the increase in viral abundance in seawater were also seen after coral treatment with mitomycin C, an antibiotic commonly used to induce the lytic cycle in latent viral infections (Figure 5B). TEM analysis of sun-screen-treated corals showed the presence of virus-like particles (VLPs) around and inside the zooxanthellae. The VLPs were round-hycosahedral in shape and 50–130 nm in size (Figure 6). No viruses were encountered either inside or outside the zooxanthellae in control samples. All these results indicate that sun-screens caused coral bleaching by inducing the lytic cycle in symbiotic zooxanthellae with latent viral infections.

Causative agents (mostly bacteria and fungi; Rosenberg et al. 2007) have been isolated and characterized for only 6 of more than 20 coral diseases described in natural environments. To date, viruses have been found in cells of about 50 algal species, representing nearly all major algal classes. This suggests that viruses have a significant role in algal ecology (Brussard 2004). There are, however, only a few studies on viruses infecting zooxanthellae: viruses were encountered in heat-shocked or UV-treated zooxanthellae of *Pavona danai*, *Acropora formosa,* and *S. pistillata*, suggesting the presence of latent viral infections (Davy et al. 2006; Lohr et al. 2007). All our samples from different areas of the world showed viral lytic cycles after treatment with sunscreens and other inducing factors. The results of the present study and these data from the literature indicate that latent infections are common in symbiotic zooxanthellae.

Viruses have a key role in population dynamics and in community composition and diversity of marine bacterioplankton and phytoplankton (Brussard 2004; Suttle 2005) Viruses also contribute significantly to horizontal gene transfer, and can influence the pathways of energy and material flow in aquatic ecosystems, with important implications for global biogeochemical cycles (Fuhrman 1999). The results presented here provide new insights into the functional and ecological role of aquatic viruses and indicate that induction of the lytic cycle in zooxanthellae with latent infection represents an important factor contributing to coral bleaching.

Recent studies have reported that pesticides, hydrocarbons, and other contaminants can cause coral bleaching (Brown 2000; Douglas 2003). We suggest that these factors, which also have the potential to induce the viral lytic cycle in microorganisms or algae with latent infections (Cochran et al. 1998; Danovaro and Corinaldesi 2003; Davy et al. 2006; Jang and Paul 1996) could act synergistically with sun-care products, thereby increasing the frequency and extent of coral bleaching.

Our results indicate that sunscreens promoting lytic cycle in viruses can cause coral bleaching. Because human use of tropical ecosystems and coral reef areas is progressively increasing, we predict that the impact of sun-screens on coral bleaching will grow considerably in the future on a global scale. Actions are therefore needed to stimulate the research and utilization of UV filters that do not threaten the survival of these endangered tropical ecosystems.

## Correction

In Table 2, the log *K*_ow_ value for 2-ethyl-hexyl salicylate has been corrected from “NA” in the original version published online to “6.02.”

## Figures and Tables

**Table 1:** Experiments on hard-coral species treated with different sunscreens and sunscreen ingredients.

Ocean	Reef area	Reef water temperature (°C)	Treatments	Sun protecting factor	Quantity [μl/L (%)]^a^	Species	No. of experimental sets	Bleaching initiation (hr)	Bleaching rate [hr (%)]^b^	Zooxanthellae released (%)
Pacific	Celebes Sea, Indonesia	28, 30^c^	Sunscreen brand 1	15	100	*Acropora divaricata*	6	ND	24 (81, 95)	ND
			Sunscreen brand 1	15	10	*A.divaricata*	6	ND	36 (ND)	ND
			Nutrients		100^d^	*A. divaricata*	6	No bleaching	No bleaching	ND
			Controls			*A. divaricata*	6	No bleaching	No bleaching	ND
Atlantic	Caribbean Sea, Mexico	28	Sunscreen brand 2	8	10	*Acropora cervicornis*	3	18	36 (84)	87
			Controls			*A. cervicornis*	3	No bleaching	No bleaching	3
			Sunscreen brand 2	8	10	*Millepora complanata*	3	24	36 (35)	10
			Controls			*M. complanata*	3	No bleaching	No bleaching	2
Indian	Red Sea, Egypt	24	Sunscreen brand 1	8	33	*Acropora* sp.	3	24	48 (81)	44
			Sunscreen brand 1	15	33	*Acropora* sp.	3	24	48 (89)	30
			Controls			*Acropora* sp.	3	No bleaching	No bleaching	1
			Sunscreen brand 1	15	33	*Stylophora pistillata*	3	nd	48 (65)	ND
			Controls			*S. pistillata*	3	No bleaching	No bleaching	ND
			BMDBM		33 (2)	*Acropora* sp.	3	No bleaching	No bleaching	13
			MBC		33 (3)	*Acropora* sp.	3	24	48 (63)	10
			OCT		33 (6)	*Acropora* sp.	3	No bleaching	No bleaching	3
			EHS		33 (5)	*Acropora* sp.	3	No bleaching	No bleaching	3
			OMC		33 (6)	*Acropora* sp.	3	2	24 (91)	86
			BZ		33 (6)	*Acropora* sp.	3	24	48 (86)	83
			BP		33 (0.5)	*Acropora* sp.	3	24	48 (84)	90
			PG (solvent)		33	*Acropora* sp.	3	No bleaching	No bleaching	16
Indian	Andaman Sea, Thailand	25^e^	Sunscreen brand 3	8	50	*Acropora pulchra, Acropora aspera, Acropora intermedia, Acropora sp.*	15	24	48–62 (74–88)	88–95
			Controls			*A. pulchra, A. aspera, A. intermedia, Acropora sp.*	15	No bleaching	No bleaching	1–2
			MBC		50 (3)	*A. pulchra*	3	48	62 (95)	95
			OMC		50 (6)	*A. pulchra*	3	48	96 (91)	90
			BZ		50 (6)	*A. pulchra*	3	48	96 (93)	84
			BP		50 (0.5)	*A. pulchra*	3	48	96 (90)	79

Abbreviations: BMDBM, 4-*tert-*butyl-4-methoxydibenzoylmethane; BP, butyl paraben; BZ, benzophenone-3; EHS, ethylhexylsalicylate; MBC, 4-methylbenzylidene camphor; ND, not detected; OCT, octocrylene; OMC, ethylhexylmethoxycinnamate; PG, propylene glycol.

a Percentage concentrations of the filters allowed in sunscreen formulations in both American and European markets.

b Bleaching rates measured as percentage chromatic dissimilarity with the coral used as a control (CMYK) at different experiment times (hr).

c Temperature in outdoor aquarium.

d Concentrations of nutrients relative to added sunscreen are calculated on the ratio of organic carbon to total nitrogen and phosphorous (wt:wt) of 31:2:1.

e Local temperature during the experiment was below average season values.

**Table 2:** Physicochemical properties of the UV filters.

Chemical name (INCI name)	Key^a^	Chemical structure	Molecular weight (g/mol)	Water solubility (mg L^–1^) at 25°C	Log *K*_ow_^b^	λ_max_
2-Hydroxyl-4-methoxybenzophenone (*benzophenone-3*)	BZ	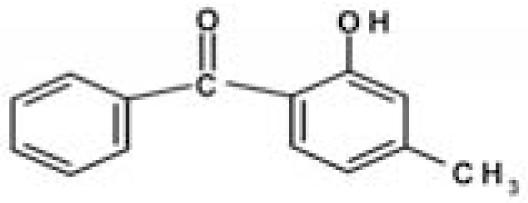	228.25	68.56	3.52	286
4-*tert*-Butyl-4’-Methoxydibenzoyl methane (*butyl methoxydibenzoylmethane*)	BMDBM	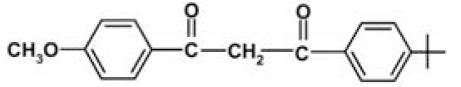	310.39	1.52	2.41	355
2-Ethylhexyl-4-methoxycinnamate (*ethylhexylmethoxycinnamate*)	OMC	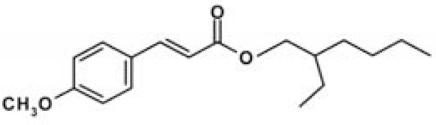	290.41	0.15	5.80	305
2-Ethylhexyl 2-cyano-3,3-diphenylacrylate (*octocrylene*)	OCT	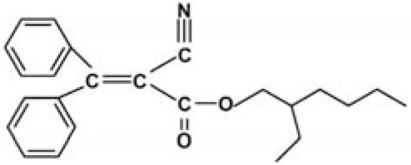	361.49	1.3	6.88	303
2-Ethylhexyl salicylate (*ethylhexyl salicylate*)	EHS	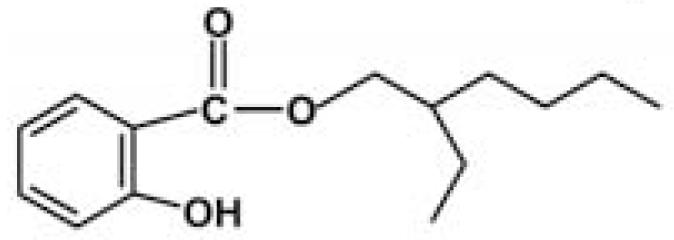	250.37	NA	6.02	305
3-(4’-Methylbenzylidene) camphor (*4-methylbenzylidene camphor*)	MBC	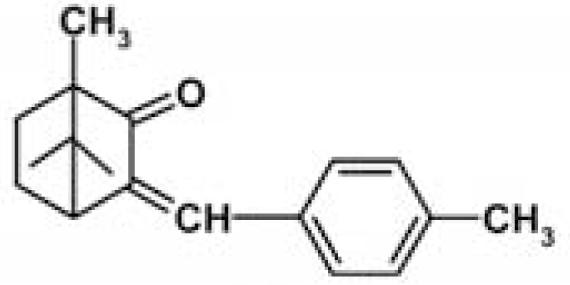	240.35	0.57	5.47	300
Butyl *p*-hydroxybenzoate^c^ (*butylparaben*)	BP	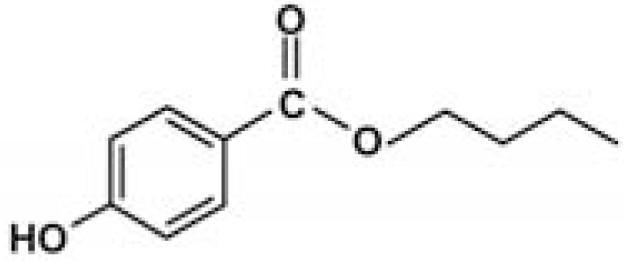	194.23	207	3.57	253

Abbreviations: INCI, International Nomenclature for Cosmetic Ingredients; NA, not available.

a Key abbreviations adopted in this paper. For acronym definitions,” see Table 1.

b Octanol/water partition coefficient.

c This is a preservative, not a UV filter.

**Table 3:** Shifts in the percentage contribution of the different coral color components [cyan, magenta, yellow, black (CMYK)] that occurred during the experiments (addition of sunscreen and sunscreen ingredients).

	Coral color shift^a^					
Treatments	C	M	Y	K	Bleaching	Significance^b^
Control	0	2	3	0	NV	NS
Sunscreen	19	25	17	33	Visible	****
BMDBM	6	22	12	33	NV	**
BZ	6	24	7	43	NV	**
OMC	13	37	23	53	Visible	***
OCT	7	23	18	39	NV	**
EHS	6	20	7	38	NV	NS
MBC	8	17	5	37	NV	**
BP	9	32	33	29	Visible	***

Abbreviations: NS, none of the four variables is significant; NV, nonvisible bleaching. For acroynm definitions under “Treatment,” see Table 1.

a Shift estimated as the average of 20 measurement points of the four colorimetric variables (CMYK).

b Significance (*p* < 0.05) of each variable calculated by ANOVA; number of asterisks indicate the number of significant variables.
